# How well do elderly patients with cervical cancer tolerate definitive radiochemotherapy using RapidArc? Results from an institutional audit comparing elderly versus younger patients

**DOI:** 10.3332/ecancer.2014.484

**Published:** 2014-11-20

**Authors:** Santam Chakraborty, M Geetha, Sampada Dessai, Vijay M Patil

**Affiliations:** 1 Department of Radiation Oncology, Malabar Cancer Centre, Thalassery, Kerala 670103, India; 2 Department of Surgical Oncology, Malabar Cancer Centre, Thalassery, Kerala 670103, India; 3 Department of Clinical Hematology and Medical Oncology, Malabar Cancer Centre, Thalassery, Kerala 670103, India

**Keywords:** uterine cervical neoplasms, aged, chemoradiation, intensity-modulated radiotherapy, toxicity

## Abstract

**Purpose:**

Elderly patients (65 or older) with cervical cancer often receive suboptimal radio-chemotherapy. Intensity-modulated radiotherapy (IMRT) may improve tolerance to treatment in this setting. This study was designed to compare the treatment-related toxicities and compliance with treatment in patients of cervical cancer treated definitively with RapidArc IMRT in our institute.

**Methods and materials:**

The treatment records of all patients treated with RapidArc IMRT between April 2012 and April 2014 were reviewed, retrospectively. Prospectively collected data regarding treatment toxicity (CTCAE 4.0), treatment outcomes and parameters related to treatment compliance were compared amongst two age groups (< 65 and ≥ 65 years). The results of 66 patients were identified, of whom 23 were found to be ≥ 65 years age. All patients completed planned external beam radiotherapy. However, significantly fewer patients in the elderly group received concurrent chemoradiation (98% versus 65%, *p* < 0.001). Old age (median 75 years, IQR: 74–78 years) was the commonest cause for non-receipt of chemotherapy. Incidence of grade 3 haematological toxicities (26.7% versus 16.7%) and gastrointestinal toxicity (16.7% versus 13.3%) were not significantly different between the two groups. Other treatment-related toxicities, breaks, treatment duration and early outcomes were also not significantly different between the two age groups.

**Conclusions:**

The use of IMRT did not result in excess toxicities in the elderly population and was associated with equivalent compliance to treatment. Concurrent chemoradiation can be safely combined in elderly patients with perfect organ function and performance status.

## Introduction

Cervical cancer is the commonest gynaecological malignancy encountered in India with a reported age-standardized incidence rate of 30.7 per 100,000 [1]. The data from various hospital-based cancer registries (HBCR) of India show that the cervical cancer is a disease of the younger population with the mean age of presentation ranging between 50 and 56.7 years [1]. Patients aged 65 or above account for around 15% of all patients in these HBCRs.

As can be anticipated, data regarding the optimal management of the cervical cancer in the elderly are limited. Wright *et al* found that elderly patients with cervical cancers treated in the United States had a significantly poorer survival rate as compared to younger patients [2]. In their analysis, elderly patients were more likely to present with advanced stage, non-squamous cell cancers and underwent surgery less frequently. Such patients are also underrepresented in clinical trials, accounting for only 3–5% of patients recruited in clinical trials for cervical cancer [3, 4].

Volumetric modulated arc therapy (VMAT) is a form of intensity-modulated radiotherapy (IMRT) initially described by Otto *et al* [5]. The commercial implementation of VMAT delivery on the Varian platform is known as RapidArc. It allows the delivery of highly conformal radiotherapy in a single or double gantry rotation with simultaneous dynamic modulation of multileaf collimator (MLC) speed as well as dose rate. Cozzi *et al* have shown that the use of VMAT (RapidArc) can result in better organ-at-risk sparing with equivalent target volume coverage as compared to fixed field IMRT [6].

Despite the higher burden of cervical cancers in India, few data exist on the treatment tolerance and toxicities encountered in elderly Indian patients with cervical cancers. Concerns have been raised regarding the poor tolerance of chemoradiation in our patients due to the high prevalence of anaemia, malnutrition, and anticipated higher toxicity [4]. Hence, the objective of the present study was to compare the treatment tolerance, compliance, toxicities, and early outcomes in the elderly (≥ 65 years) against the non-elderly group of patients (< 65 years) receiving definitive external beam radiotherapy (± chemotherapy) with RapidArc IMRT.

## Materials and methods

The treatment records of all patients treated with definitive RapidArc IMRT for cervical cancer between April 2012 and April 2014 were audited. All patients were started on treatment after a multi-disciplinary consultation which included evaluation by a trained gynaecological oncologist. All patients underwent a standard staging workup which included clinical evaluation, chest radiography, cystoscopy, and computed tomography of the abdomen and pelvis. At present, a comprehensive geriatric assessment is not performed in our hospital due to resource constraints.

### Simulation

Patients were simulated in the supine position. Computed tomography (CT) images were acquired in from the level of the top of the diaphragm to the mid-thigh. All patients received oral and rectal contrast as per departmental protocol. 70 ml of non-ionic intravenous contrast was administered during the acquisition process, subject to normal renal functions. Images were transferred to the Eclipse Treatment Planning System version 10 (Varian Medical System, Palo Alto, California, USA). From May 2013 onwards, one full-bladder and one empty-bladder scan were acquired in order to generate the internal target volume (ITV). A bowel preparation protocol consisting of a mild laxative for 2 days prior to and an enema on the day of the scan was utilized to minimize rectal distension.

### Image segmentation

The clinical target volume (CTV) included the entire uterus, cervix, and at least the upper two-thirds of the vagina along with the common iliac, internal iliac, external iliac, presacral, and obturator group of lymph nodes. Nodal CTVs were delineated according to the guidelines proposed by Taylor *et al* [7], while primary tumour volumes were delineated using the guidelines proposed by Lim *et al* [8, 9]. The nodal GTV included any enlarged pelvic/paraaortic lymph nodes that were greater than 1.5 cm in the short axis dimension or had intranodal necrosis. As PET-CT was not available in the institution, patients were advised PET-CT only if para-aortic lymphadenopathy was detected to rule out distant metastases. The details of the target delineation protocol followed are given in Table 1. Organs at risk, including the bladder, rectum, bowel loops, bilateral femoral, and pelvic bone marrow, were delineated. The bladder and rectum were delineated as solid organs including the entire wall. The rectum was delineated from the anal verge until the sigmoid flexure. Individual loops of the small and large bowel were delineated as a single structure labelled as the bowel loops. Initially, pelvic bone marrow was delineated as a single structure through bone auto-segmentation available in the treatment planning system. Subsequently, the generated contours were checked slice-by-slice and edited manually as well as with post-processing to fill in cavities in the contours and remove contours outside the bone marrow.

Until April 2013, an ITV was generated by expanding the contours of the uterus and cervix 2 cm anteriorly and 1 cm in other directions [10]. Since May 2013, after a departmental review of the contouring practice two scans, one full-bladder and one empty-bladder scan were taken for all patients. Both scans were registered using rigid registration to demarcate the ITV (Table 1). The entire nodal CTV and ITV were expanded by a margin of 1 cm axially and 1.5 cm cranio-caudally as per institutional protocols to generate the planning target volume (PTV). In patients receiving a simultaneous integrated boost (SIB) to the involved lymph nodes the nodal GTV was expanded by a same PTV margin to generate a high-dose PTV. The decision to give an SIB was left to the discretion of the treating physician.

### Treatment planning

A dual-arc monoisocentric coplanar RapidArc with total of 177 control points was used for all pelvic IMRT plans. In patients requiring para- aortic nodal radiation, two isocentres were utilized with one overlapping arc for each arc. Both arcs were full 360° arcs. The arc geometry tool of the Eclipse treatment planning system was used to set the arc isocentre, which was then visually verified and adjusted as required. Collimator rotation was kept at 30° for the clockwise arc and 330° for the counter-clockwise arc. The field size in the X direction was kept at a maximum of 15 cm.

Optimization that was done using the Progressive Resolution Optimizer (PRO) algorithm version 10 (Varian Medical System, Palo Alto, California, USA). A dose of 45–50.4 Gy in 25–28 fractions was prescribed for the entire PTV. Patients receiving SIB received a boost dose ranging between 55 and 61.6 Gy to the high-dose PTV. The optimization objectives included coverage of 95% of the PTV with 100% of the prescribed dose and overdose to be less than 115% to less than 0.03 cm^3^ volume. Organ-at-risk dose constraints included the following:

- Priority 1: bowel loops: V_45_ less than 149 cm^3^ (V_45_ = volume receiving 45 Gy)
- Priority 2: bone marrow: mean dose < 40 Gy
- Priority 3: bladder and rectum: D2 cm^3^ ≤ 50.4 Gy.

Normal Tissue Objective (NTO)—a parameter that limits dose spillage as a function of distance from the PTV, [11] was used during the optimization to constraint dose spillage into normal tissues outside the PTV. Optimized plans were calculated using the anisotropic analytical algorithm (AAA) with a dose grid size of 2.5 mm. Plans were iteratively re-optimized until all planning goals were met. Specific control structures were created to reduce overdose or improve doses in areas of underdose. Dose spillage outside PTV was also corrected using control structures where required. In addition, we also changed the priorities for organs at risk when dose constraints were not met in the first round of optimization. Usually, four to five plan iterations were required for each patient. Plan normalization was done to the target mean dose. The homogeneity index and conformity index were defined as per the ICRU 83 definition:

- homogeneity index = D2 (minimum dose to 2% volume) − D98 (minimum dose to 98% volume)/D50 (median dose)
- conformity index = volume of the PTV receiving the prescribed dose/volume of PTV.

### Plan quality assurance and treatment delivery

Prior to the treatment delivery plan, quality assurance was performed using an Octavius II phantom with a PTW 729 ionization chamber (Physikalish-Technische Werkstätten-Freiburg, Freiburg, Germany) array. The verification plan consisted of two arcs delivered to a single isocentre. Gamma evaluation was done using Low’s method [12] with a dose difference of 3% and distance to agreement of 3 mm. Results were analysed on the Verisoft software version (Physikalish-Technische Werkstätten-Freiburg, Freiburg, Germany).

Patients were treated 5 days a week with one fraction each day. A standard bladder-filling protocol was followed in which patients were instructed to drink 1000 cm^3^ of water daily and wait for 1 h after voiding before the radiotherapy. Patients who underwent concurrent chemoradiation received Cisplatin in the doses of 40 mg/m2 weekly. Cone beam CT was used for detection and correction of setup errors initially for the first three fractions and then twice weekly or more frequently as indicated. Patients were reviewed for toxicity weekly, and toxicity was recorded prospectively on the treatment charts. Radiation-related toxicity was scored as per the common terminology criteria for adverse events (CTCAE version 4.02) acute toxicity criteria. The highest-grade toxic event noted during the entire duration of treatment (both external beam radiotherapy and brachytherapy) are reported in the present study.

Patients were assessed for brachytherapy 1 wk after completion of external beam radiotherapy. Three high-dose-rate (HDR) brachytherapy insertions were planned with a prescribed dose of 7 Gy to point A one week apart (cumulative EQD2 = 79.3 Gy10) [13]. Until June 2013, conventional X-ray based planning was performed with rectal and bladder point doses being constrained to 75–80% of the prescribed point A doses. Subsequently, all patients who had CT-based volumetric planning were adopted, and doses were constrained as per the GECESTRO recommendations [14] (total rectal and sigmoid EQD2 < 75 Gy3 and bladder EQD2 < 90 Gy_3_).

### Follow-up

After the treatment, the patients were evaluated clinically monthly for the first three months and then at two-month intervals thereafter for the first year. After the first year, the patients were followed up at three-month intervals for the second year and six-month intervals thereafter. Clinical examinations, including local and per-rectal examination, were the mainstay of evaluation, with appropriate imaging performed only if clinically indicated. Tissue confirmation of recurrence was obtained in the setting of a suspicious recurrence.

### Statistical analysis

The audit was approved by the institutional ethics committee and requirement for informed consent waived in view of the retrospective nature of the study. Statistical analysis was performed using SPSS (Statistical Package for Social Sciences, SPSS Inc, Delaware, Chicago, USA) version 16. The patients were divided into two categories using the age of 65 years as a cutoff (< 65 years and ≥ 65 years).

The chi-square test was used for comparing frequencies in the two groups for categorical variables. Independent sample *t* test was used for comparing the means of continuous variables. The duration of locoregional control, progression-free survival, and overall survival were calculated from the date of registration to the date of the event or last follow-up. For the calculation of progression-free survival, patients without demonstrable locoregional/distant disease were censored. The patients without demonstrable disease locally or in pelvic lymph nodes were censored in the calculation for locoregional recurrence-free survival. Patients alive with or without disease were censored at the last follow-up for the calculation of the overall survival. The Kaplan–Meier method was used for the estimation of survival and log-rank test was used to compare the survival between the age categories. *P* values less than 0.05 were taken as significant and bonferroni corrections were applied for multiple comparisons.

## Results

### Patient population

During the specified period, 99 patients were treated for cervical cancer in our institute with curative intent. Out of this, 23 were excluded as they were treated with 3DCRT, while ten had received post-operative radiotherapy. Thus, 66 patients were eligible for analysis based on the selection criteria mentioned above.

Out of these 66 patients, 23 patients (34.8%) were found to have age of 65 years or more. In this group of 23 patients, 16 patients were aged < 75 years and seven patients ≥ 75 years. The baseline demographic and disease-related variables are shown in Table 2.

### Plan and dosimetric results

The median total monitor units (MU) delivered for the RapidArc plans were 568 (interquartile range: 502.5–633.5). Table 3 shows the dosimetric results obtained for the PTV as per the age category. The average mean doses for the bone marrow, rectum, bladder, small bowel, and femoral heads were 36.1 Gy, 49.1 Gy, 46.1 Gy, 29.0 Gy, and 22.9 Gy, respectively, for the entire population with no significant difference noted as per the age category (Table 4).

### External beam radiotherapy

The median duration to the start of radiotherapy from the date of registration was 26 (Interquartile Range: 18.5–33.5) days with no significant differences noted across age categories. All patients completed the planned course of external beam radiotherapy. The details of treatment used and the compliance to the treatment are shown in Table 5.

### Concurrent chemoradiation

Eight of 23 patients ≥ 65 years did not undergo concurrent chemoradiation. One patient did not undergo concurrent chemotherapy due to abnormal renal function (Creatinine clearance < 40). In the remaining seven patients who did not undergo chemoradiation, the reason was old age. In these seven patients, the median age was 75 years (Interquartile Range: 74–78 years). One patient < 65 years did not undergo chemoradiation due to abnormal renal function.

### Concurrent chemoradiation interruptions

Of the 57 patients started on concurrent chemotherapy, 26 could not complete the planned course of chemotherapy. Of these, 18 (42.8%) were aged < 65 years and eight (53.3%) were aged ≥ 65 years. In the eight patients with age ≥ 65 years, the reasons for chemotherapy interruption were haematological, gastrointestinal, and renal toxicity in four (50.0%), three (37.5%) and one (12.5%) patients, respectively. In the 18 patients with age < 65 years, reasons for chemotherapy interruption were haematological, gastrointestinal, and renal toxicity in nine (50%), four (22.2%), and three (16.6%) patients, respectively. In two patients, chemotherapy was interrupted due to lower respiratory tract infection. There were no statistically significant association between the age group and these parameters by chi-square test.

The median-delivered chemotherapy dose intensity in patients < 65 years was 88.2% in patients whose chemotherapy was completed as planned (*n* = 27) and 56.6% in whom it was interrupted prematurely (*n* = 18). In patients ≥ 65 years, the median-delivered dose intensity was 90.6% in patients undergoing chemotherapy as planned (*n* = 7) and 55.6% in whom it was interrupted prematurely (*n* = 8). There were no statistically significant association between the age group and these parameters by chi square test.

### Brachytherapy

Three patients aged > 65 years did not undergo brachytherapy. Among these three patients, one was not given concurrent chemoradiation initially due to renal dysfunction. Two patients, both aged > 65 years, refused brachytherapy, while one patient underwent supplementary external beam radiotherapy due to poor response and distorted anatomy after external beam radiotherapy.

In the 63 patients who underwent brachytherapy, tandem ovoid applicator was used for intracavitary brachytherapy in 56 (88.9%) patients. Vaginal cylinders with intrauterine tubes were used in two (3.1%) patients, and template-based Interstitial brachytherapy was used in five (7.9%) patients. Thus, 64 (96.9%) patients completed planned treatment.

### Treatment-related toxicities

All patients were assessable for toxicity. Table 6 shows the number and proportion of patients experiencing various toxicities. In the 66 patients, there were two patients with grade IV toxicities—one with grade IV hyponatremia and one with grade IV hypomagnesemia. No deaths were observed due to treatment-related toxicities and the 30-day mortality rate was 0%. Any grade 3–4 toxicities were seen in 19 (44.1%) patients < 65 years age and in eight (34.7%) patients who were ≥ 65 years age (*P* = 0.42). Patients with grade 3–4 toxicities did not have a significant prolongation in the overall treatment time or gap between external radiation and brachytherapy as compared to patients without toxicities.

On restricting the analysis to the 57 patients who had received concurrent chemoradiation, there was no difference in the incidence of any grade 3–4 toxicities in patients younger than or older than 65 years (42.9% versus 40% respectively, *P* = 0.38). In addition, there was no difference in the incidence of any grade 3–4 gastrointestinal toxicities (16.7% versus 13.3%, *P* = 0.76) or grade 3–4 haematological toxicities these 57 patients (26.7% versus 16.7%, *P* = 0.40) across the age category.

### Treatment outcomes

Local residual disease was documented at three months post-radiation in seven patients. Three out of 43 (7%) patients aged < 65 and four out of 22 (14.9%) patients aged age ≥ 65 years had local residual disease (*P* = 0.23). The median follow-up was 11.5 months, with no significant difference according to the age category. At last follow-up, 12 patients (18.2%) of 66 had disease progression. The patterns of failure included locoregional disease alone in six (9.1%), distant metastases in five (7.57%), and one had both distant metastases and locoregional recurrence (1.5%).

One-year progression-free survival in patients < 65 and ≥ 65 years was 95% (95% confidence intervals: 100.0–78.2%) and 75% (95% CI: 100.0–62.0%), respectively. One-year locoregional recurrence-free survival was 95%(100.0–82.5%) and 88% (100.0–76.0%), respectively. One-year overall survival in both groups was 100%. There was no significant difference in any of the outcome-related parameters among the two age groups.

## Discussion

In our hospital, which is located in the Malabar region of Northern Kerala, elderly patients (65 years or more) account for almost 27% of the cervical cancer patients [15]. This is in contrast to the consolidated report from the Hospital-Based Cancer Registry (HBCR), where elderly patients accounted for approximately 10–12% of the cervical cancer cases in most cancer registries [1]. This is possibly secondary to the effect of the ongoing demographic transition in Kerala [16], as elderly patients accounted for 28% of all cervical cancers in the Thiruvananthapuram-based HBCR also, which is located in the state of Kerala [1].

In the present study, the common iliac nodal CTV was drawn up to the bifurcation of the aorta. This was in accordance with the guidelines given by Taylor *et al* [7], but not in accordance with the nodal CTV delineation guidelines proposed by Small *et al* [17]. As shown by Rai *et al* recently out of the nine patients with failures above the field borders, five had failures located between the L4-L4 interspace and the aortic bifurcation [18]. There was no effort made to treat elderly patients with more conservative volumes as is apparent in the fact that the PTV volumes did not differ significantly between the two groups.

Despite the use of a larger PTV volume, the tolerance to the definitive radiotherapy was good. Breaks greater than 3 days were required during EBRT for only 6% of the patients and all patients received the planned dose of external radiation. Noteworthy is the fact that the tolerance to the treatment was similar across both age groups. This is in contrast to a series of 105 elderly patients where conventional radiotherapy techniques were used, and approximately 13% of the patients could not complete radiotherapy secondary to toxicities or other causes [19].

In contrast to Kunos [20] and Laurentius *et al* [21], who have reported higher haematological toxicity in elderly patients undergoing chemoradiation, we found no increased incidence of haematological toxicity in elderly patients who received chemoradiation. Our rates of grade 3–4 gastrointestinal and genitourinary toxicity were also lower than other studies reporting outcomes of definitive radiation in elderly patients which employed conventional radiotherapy [19, 21].

Furthermore, most of the haematological toxicity observed was in the form of anaemia which is similar to the experience reported by Mahantshetty *et al* [22]. The incidence of grade 3–4 leucopenia was similar or better than other studies employing IMRT, although a slightly higher incidence of grade 3 neutropenia was observed [22, 23]. In the present study, we have reported the maximum grade of toxicity across entire treatment including brachytherapy. This is important as several patients have worsening haematological toxicity after completion of chemoradiation and during brachytherapy, which is not reported if only toxicity during external beam radiotherapy is reported.

The better tolerance to EBRT possibly helped in the higher compliance with brachytherapy. As shown by Park *et al*, 18% of elderly patients with carcinoma of the cervix did not receive brachytherapy [19]. In the SEER analysis conducted by Sharma *et al* [24], less than 50% of the patients older than 70 years received some form of brachytherapy. Similarly, Laurentius *et al* found that 36% patients did not undergo brachytherapy in their retrospective study of patients of cervical cancer treated with radiochemotherapy.

The disparity in the delivery of chemoradiation among age groups was significant as was the lesser number of cycles that were administered. In the elderly group of patients, all four patients who had chemotherapy interrupted due to haematological toxicity had grade II leucopenia. This is a probably a reflection of the increased cautioness on the part of our oncologists in treating elderly patients with concurrent chemoradiation.

A reluctance to prescribe chemoradiation in elderly has been reported in Western series as well. In an audit of 1900 patients from two regions of the Netherlands, it was found that chemoradiation was delivered to only 2% of the patients older than 70 years [25]. Similarly, in another study by Goodheart *et al* [26], 44% of the patients older than 65 years did not receive concurrent chemoradiation. While we did not find any significant differences any of the toxicities encountered in elderly versus non-elderly patients, given the retrospective nature of the comparison and the limited number of patients the results need to be interpreted cautiously.

The inadequate representation of elderly patients in clinical trials conducted in cervical cancers makes it difficult to generate evidence-based recommendations for their treatment. In a recent meta-analysis of 13 randomized-controlled trials [27], no age group was found to benefit more or less from concurrent chemoradiation, suggesting that age should not be a criterion used forego chemoradiation in this population.

This study is limited by its short follow-up, modest sample size, and retrospective design. In addition, no dedicated geriatric assessment was done for the elderly patients. However, patients selected for this series were generally fit as most patients had PS 0 -1 and a normal BMI (Table 2). Although treatment-related toxicities were recorded prospectively, we cannot rule out a higher burden of toxicities as patient-reported toxicity, and the quality of life data was not collected. However, the overall low incidence of severe toxicity, good compliance and acceptable early outcomes are encouraging.

## Conclusion

Elderly patients seem to tolerate RapidArc IMRT-based radiotherapy (± chemotherapy) well without an increase in treatment-related toxicities as compared to younger patients. The low rates of radiation-related toxicities as compared to historical studies employing conventional radiotherapy in this age group may allow increased the utilization of concurrent chemoradiation. In the future, our department plans to prospectively study the utility of comprehensive geriatric assessment to help decision making regarding the incorporation of chemoradiation in elderly patients.

## Figures and Tables

**Table 1.** The present departmental guidelines followed for delineation of the nodal CTV in the patients. In all cases, the nodal CTVs were trimmed from the muscles, bones, bladder, rectum, and small bowel. *N.B - Prior to May 2013, the entire primary tumour CTV was expanded by 20 mm anteriorly and 10 mm in other directions to generate an ITV (internal target volume).

Primary Tumour	Entire uterus, adenxa, cervix, parametria and upper 2/3 of vagina. The parametria were contoured bilaterally extending laterally up to the pelvic side walls. Superiorly, the parametria was contoured until the level at which the small bowel or the round ligaments were visible. In patients with vaginal involvement greater than upper 1/3, the entire vagina up to the introitus was contoured as the primary tumour CTV.
ITV*	The primary tumour CTV was delineated separately on the full-bladder and empty-bladder CT scans. These scans were then co-registered using automatic rigid registration (along with manual corrections when required). Both CTVs were boolean (using an OR operator) to generate the ITV.
Common Iliac Nodal CTV	7 mm margin around common Iliac vessels with extension of the posterior and lateral border to the recess between psoas muscle and vertebrae. Superiorly, the contour started from the level of the aortic bifurcation.
External Iliac Nodal CTV	7 mm margin around external iliac vessels with additional extension of 10 mm in the anterolateral direction along the iliopsoas muscle to include the lateral external iliac group of lymph nodes. The caudal extent of the contours was at the slice at which the femoral heads were visible.
Internal iliac Nodal CTV	7 mm margin around the internal iliac vessels.
Presacral Nodal CTV	1 cm strip of tissue in front of the sacrum joining the internal iliac vessel contours. Inferior extent up to the S2–S3 junction.
Obturator Nodal CTV	1.5 cm strip joining the external and internal iliac nodal CTVs. The caudal extent of the contours extend up to the level of the pubic symphysis.

**Table 2.** The demographic- and disease-related variables in the population. For categorical variables percentages in parentheses represent the percentage within the age category. *P* values reported are from Chi-square test for categorical variables and *t* test for continuous variables. SCC = Squamous Cell Carcinoma, AC = Adenocarcinoma, BMI = Body Mass Index, IQR = Interquartile Range. *P* value < 0.005 are taken as significant (indicated with #) after Bonferroni correction.

Variable	Value	Age < 65 (*n* = 43)	Age ≥ 65 (*n* = 23)	*P* value
Age	Median (IQR)	55 (48–60)	70 (66–75)	< 0.001
Performance status	0–1	41 (95.3%)	21 (91.3%)	0.38
	2–3	2 (4.6%)	2 (8.7%)	
Co-morbidity	Absent	24 (55.8%)	9 (39.1%)	0.15
	Present	19 (44.2%)	14 (60.9%)	
Pathology	SCC	39 (90.7%)	22 (95.7%)	0.13
	AC	4 (9.3%)	1 (4.3%)	
BMI	< 18.5	7 (16.3%)	5 (21.7%)	0.79
	18.5–25	24 (55.8%)	14 (56.5%)	
	> 25	12 (27.9%)	5 (21.7%)	
Pretreatment haemoglobin (g/dl)	Median (IQR)	12.1 (10.7–13.0)	11.5 (10.3–12.4)	0.13
Pretreatment anaemia (< 10 g/dl)	Yes	4 (9.3%)	4 (17.4%)	0.28
	No	39 (90.7%)	19 (82.6%)	
Creatinine clearance (ml/min)	Median (IQR)	93 (77–106)	74 (59–97)	0.23
Primary size (cm)	Median (IQR)	5 (4–5)	5 (3–5)	0.65
Bulky disease	Yes	23 (53.5%)	13 (56.5%)	0.99
	No	20 (46.5%)	10 (43.5%)	
Stage (FIGO 2010)	IB	2 (4.6%)	2 (8.7%)	0.84
	IIA–B	20 (46.5%)	10 (43.4%)	
	IIIA–B	21 (48.9%)	11 (47.8%)	
Pelvic lymphadenopathy	Yes	20 (46.5%)	9 (39.1%)	0.61
	No	23 (53.5%)	14 (60.9%)	
Para-aortic lymphadenopathy	Yes	6 (14.0%)	1 (4.3%)	0.40
	No	37 (86%)	22 (95.7%)	

**Table 3.** The PTV volume and dosimetric results for the PTV in the two age categories. D98%: Minimum dose to 98% volume, D50%: Median dose, D2%: Minimum dose to 2% volume. *P* value < 0.007 are significant after Bonferrroni correction.

	Age < 65 (*n* = 43)		Age ≥ 65 (*n* = 23)		*P* Value
	Mean	Std Dev	Mean	Std Dev	
Volume	1610.9	395.6	1677.6	434.6	0.61
D98%	48.9	1.8	48.8	1.6	0.50
D2%	53.9	1.5	53.9	1.3	0.61
Median	51.7	1.6	51.9	1.3	0.61
Mean	51.7	1.6	51.7	1.2	0.30
Homogeneity index	0.1	0.0	0.1	0.0	0.61
Conformity index	0.9	0.1	0.9	0.2	0.30

**Table 4.** Dose volume parameters for the the normal organs at risk. V15, 30, 45, and 50 Gy represent the absolute volume receiving 15 Gy, 30 Gy, and 45 Gy, respectively, in cc. *P* values < 0.008 taken as significant after Bonferroni correction for each OAR. Dmean represents the Mean Dose in Gy. Volumes are in cm^3^.

	Age < 65 (n = 43)	Age ≥ 65 (*n* = 23)	Mean	Std Dev	*P* Value
	Mean	Std Dev			
**Bone Marrow**					
Volume	558.3	300.6	519.2	132.4	0.61
Dmean	35.9	3.0	36.5	2.9	0.90
V15	510.1	243.0	488.5	125.4	0.30
V30	371.0	181.3	355.6	112.9	0.30
V45	98.6	65.5	112.9	60.3	0.61
**Rectum**					
Volume	74.3	28.9	101.1	64.9	0.61
Dmean	49.2	3.2	48.7	4.7	0.86
V15	73.9	28.9	100.8	64.6	0.61
V30	73.2	28.5	93.9	54.6	0.61
V45	63.3	28.3	84.0	56.0	0.61
**Bladder**					
Volume	455.2	230.1	291.5	212.3	0.12
Dmean	46.0	3.4	46.3	4.2	0.61
V15	454.0	228.0	290.8	207.0	0.12
V30	428.0	203.0	285.2	175.1	0.30
V45	274.4	126.4	217.7	140.4	0.12
	**Bowel Loops**				
Volume	727.2	367.3	898.6	401.0	0.30
Dmean	29.0	4.8	29.0	5.0	0.90
V15	608.1	299.1	780.7	349.6	0.04
V30	289.2	132.3	419.3	205.6	0.04
V45	100.7	57.4	174.4	112.8	0.04
	**Femoral Head**				
Volume	160.1	38.6	132.0	25.6	0.04
Dmean	22.5	4.1	23.8	4.1	0.04
V15	122.0	134.1	103.2	36.8	0.04
V30	46.2	21.4	44.3	22.6	0.04
V45	8.9	8.5	8.4	6.2	0.04

**Table 5.** Treatment delivered and treatment compliance. Data for cycles of chemotherapy, delivered dose intensity, delivered dose intensity ≥ 80% and chemotherapy interruption are for the 57 patients who were started on concurrent chemoradiation. IQR = Interquartile range, EBRT = External Beam Radiotherapy. *P* value < 0.003 are significant (indicated with #) with Bonferroni correction.

Variable	Parameter	Age < 65 (*n* = 43)	Age ≥ 65 (*n* = 23)	*P* value
Dose EBRT (Gy)	Median (IQR)	50.4 (50.4–55)	50.4 (50.4–50.4)	0.44
Fractions	Median (IQR)	28 (28–28)	28 (28–28)	0.19
Duration of EBRT (days)	Median (IQR)	38 (37–39)	38 (37–39)	0.67
EBRT break for toxicity	Yes	10 (23.3%)	5 (21.7%)	0.89
	No	33 (76.7%)	18 (78.3%)	
Duration of break for toxicity (days)	Median (IQR)	2 (1–3)	3 (1–4)	0.36
Break > 3 days	Yes	2 (4.7%)	2(8.7%)	0.40
	No	41 (95.3%)	21 (91.3%)	
Simultaneous integrated boost	Yes	11 (25.6%)	4 (17.4%)	0.45
	No	32 (74.4%)	19 (82.6%)	
Extended field RT	Yes	6 (14.0%)	1 (4.3%)	0.23
	No	37 (86.0%)	22 (95.7%)	
Concurrent chemotherapy	Yes	42 (97.7%)	15 (65.2%)	< 0.001#
	No	1 (2.3%)	8 (34.8%)	
Cycles of chemotherapy	Median (IQR)	5 (4–5)	3 (3–4)	0.02
Delivered dose intensity	Median (IQR)	0.81 (0.58–0.90)	63.8 (0.53–0.91)	0.34
Brachytherapy	Yes	43 (100.0%)	20 (87.0%)	0.02
	No	0 (0.0%)	3 (13.0%)	
Gap between EBRT and brachytherapy (days)	Median (IQR)	8 (7–13)	8 (6–11)	0.29
Overall treatment time (days)	Median (IQR)	57 (55–66)	59 (52–62)	0.67

**Table 6.** The number of patients experiencing various toxicities during treatment (external beam radiotherapy and brachytherapy). Percentages in parenthesis show the proportion with respect to the number of patients in each group. Grade 2 toxicity not existing for hyponatremia hence grade 3 and 4 reported separately. Only two grade 4 toxicity events were recorded. *P* values less than < 0.004 taken as significant after Bonferroni correction.

Variable	Parameter	Age < 65 (*n* = 43)	Age ≥ 65 (*n* = 23)	*P* value
Haematological Toxicities				
Anaemia	Grade 2	23 (53.5%)	6 (26.1%)	0.18
	Grade 3–4	4 (9.3%)	4 (17.4%)	
Leucopenia	Grade 2	11 (25.6%)	4 (17.4%)	0.04
	Grade 3–4	5 (11.6%)	0 (0.0%)	
Neutropenia	Grade 2	4 (9.3%)	3 (13.0%)	0.05
	Grade 3–4	4 (9.3%)	0 (0.0%)	
Thrombocytopenia	Grade 2	2 (4.7%)	1 (4.3%)	0.76
	Grade 3–4	1 (2.3%)	0 (0.0%)	
Mucosal and Actinic Toxicities				
Dermatitis	Grade 2	2 (4.7%)	2 (8.7%)	0.71
	Grade 3–4	5 (11.6%)	1 (4.3%)	
Nausea & vomiting	Grade 2	12 (27.9%)	8 (34.8%)	0.81
	Grade 3–4	6 (14.0%)	2 (8.7%)	
Diarrhoea	Grade 2	12 (27.9%)	8 (34.8%)	0.92
	Grade 3–4	5 (11.6%)	2 (8.7%)	
Cystitis	Grade 2	10 (23.3%)	2 (8.7%)	0.33
	Grade 3–4	0 (0.0%)	0 (0.0%)	
Biochemical toxicities				
Acute kidney injury	Grade 2	2 (4.7%)	1 (4.3%)	0.26
	Grade 3–4	0 (0.0%)	0 (0.0%)	
Hyponatremia	Grade 3	4 (9.3%)	2 (8.7%)	0.59
	Grade 4	0 (0.0%)	1 (4.3%)	
Hypokalemia	Grade 2	0 (0.0%)	1 (4.3%)	0.23
	Grade 3–4	1 (2.3%)	2 (8.7%)	
Hypocalcaemia	Grade 2	1 (2.3%)	1 (4.3%)	0.40
	Grade 3–4	0 (0.0%)	0 (0.0%)	
Hypomagnesaemia	Grade 2	0 (0.0%)	0 (0.0%)	0.43
	Grade 3–4	1 (2.3%)	0 (0.0%)	
